# Letter to the editor regarding Unuvar BS, Yilmaz K, and Celik F: the acute effects of brace use on lower extremity performance in individuals with adolescent idiopathic scoliosis. Ir J Med Sci. 2023

**DOI:** 10.1007/s11845-024-03605-9

**Published:** 2024-01-13

**Authors:** Hans-Rudolf Weiss

**Affiliations:** Schroth Best Practice Academy, Haarbergweg 2, 55546 Neu-Bamberg, Germany

It was with great interest that I read the work of Bayram Sonmez Unuvar and colleagues entitled “The acute effects of brace use on lower extremity performance in individuals with adolescent idiopathic scoliosis.”

The content of the work focusses on the limitations that wearing a brace can have on the everyday functions of patients with adolescent idiopathic scoliosis. In principle, it is self-explanatory that wearing a trunk brace restricts the functions of daily life. Nevertheless, it makes sense to investigate this issue scientifically. However, this work falls far short of the possibilities for shedding light on the subject.

First of all, the authors report that the study included individuals between the ages of 10 and 18 years. For reasons of comparability, the inclusion criteria of the Scoliosis Research Society (age 10–14 years, Risser 0–2, angle of curvature according to Cobb 25–40°) were drawn up for studies on brace treatment [1]. Therefore, the cohort studied, with an average age of > 14 years, is well above the typical age for full-time care.

The authors also state that patients with AIS can expect a variety of complications without naming any of these complications. In contrast, it is known from the 50-year follow-up of untreated idiopathic scoliosis by Weinstein and coworkers [2] that untreated patients with this diagnosis have no significant functional limitations even in late adulthood except for a slightly increased incidence of pain (this also applies to operated scoliosis patients) and cosmetic concerns.

The authors also state that a brace can exert pressure on the spine. This is a false assumption, or at least not clearly expressed: a brace has no direct effect on the spine, as the pressure zones in the thoracic region are located on the ribs and in the lumbar region over the autochthonous back muscles. At best, a correction in the brace is produced by a corrective movement with the aim of mirroring the curvature pattern of the trunk [3].

The authors merely state Chêneau-type brace was used. The shape of the brace used is neither described further in the text nor documented in pictures. It is well known today that the international brace standard has considerable variations with success rates ranging from less than 50% to more than 90% and that this also applies to Chêneau style braces [4]. It is essential for a study on the functional limitations of the brace that the aid on which the study is based is comprehensively described in both written and pictorial form. There are long Chêneau braces and relatively short ones that restrict function so little that even gymnastic exercises can be performed with the brace [5]. The design of the Chêneau brace used is therefore essential for assessing the restrictions to be expected.

In addition to the descriptions of the individual functional tests, illustrations of these tests would also be helpful to give the reader an impression of the test setup!

The authors write: “To the authors’ knowledge, this is the first study to evaluate the effects of wearing a spinal brace on vertical jump, postural control, reach distance, and fall risk in individuals with AIS.” However, it is not immediately clear why the work was written and why these rather arbitrarily selected exercises were used for the tests.

Further down, the authors write: “Further investigations spanning an extended duration could provide a more comprehensive understanding of the sustained implications on lower extremity performance.” However, the authors do not write why a more in-depth understanding of the functional limitations should be necessary, especially as it must be assumed, even without any statistical testing, that wearing a trunk orthosis is likely to restrict the everyday function of those affected.

The following statement can also be found in the discussion: “The findings of decreased lower extremity performance associated with brace use in individuals with AIS hold significant clinical implications.” Here too, the authors fail to provide a precise explanation of the implications. The assumed restrictions on sporting activities are probably rather irrelevant if one assumes that the brace is worn for less than 20 h a day. Most sporting activities are probably possible within 4 h a day without a brace.

Under no circumstances should this study influence the decision-making of clinicians, since brace treatment has been validated in a randomized controlled trial [6] and it is now clear that high-quality brace treatment with quality-assured asymmetric Chêneau derivatives can even bring about permanent corrections of the spine and trunk [3, 4].

Thus, according to the results of this study, there is no need for any consideration, and the sentence “Clinicians need to weigh the benefits of scoliosis brace treatment against the potential limitations it poses on an individual’s physical abilities.” seems rather exaggerated in the light of the available studies.

What has been completely ignored is the question of the extent to which the brace has led to a psychological impairment of the patient’s function and what proportion of the functional limitation actually has to do with the biomechanical conditions.

In summary, it can be stated that the study cannot be considered to be of any significant relevance. It is not based on a balanced or appropriate literature review. The reader cannot deny the impression that the exaggeratedly negative diagnosis of AIS is an attempt to generate meaning. However, the fact that neither the functional tests nor the brace used are illustrated, and the latter is not even described in the text, does not correspond to good scientific practice.
